# Contamination of Wheat Flour and Processed Foodstuffs with Soybean and Mustard Allergenic Proteins

**DOI:** 10.3390/ijms26083891

**Published:** 2025-04-20

**Authors:** Mariachiara Bianco, Domenico De Palma, Antonio Pagano, Ilario Losito, Tommaso R. I. Cataldi, Cosima D. Calvano

**Affiliations:** 1 Department of Chemistry, University of Bari Aldo Moro, via Orabona 4, 70126 Bari, Italy; a.pagano21@studenti.uniba.it (A.P.); ilario.losito@uniba.it (I.L.); tommaso.cataldi@uniba.it (T.R.I.C.); 2 Interdepartmental Research Center SMART, University of Bari Aldo Moro, via Orabona 4, 70126 Bari, Italy; 3 Food Safety Lab, Via A. Santelia Architetto, 258, 70033 Corato, Italy; domenico.depalma@foodsafetylab.it

**Keywords:** allergens, soybean, mustard, mass spectrometry, flour

## Abstract

In recent years, sustainable agricultural practices in wheat cultivation have garnered significant attention, particularly those focused on minimizing pesticide and herbicide usage to safeguard the environment. One effective approach is green manuring, which entails rotating wheat with crops such as soybean and mustard to harness their natural pesticidal and herbicidal properties. While this method presents clear environmental advantages, it also poses a risk of cross-contamination, as these globally recognized allergens may unintentionally pass through wheat-based products. To protect consumers with allergies, there is an urgent need for a reliable analytical method to detect and quantify these allergenic proteins in wheat-derived foodstuffs. In this study, we assessed various protein extraction protocols to optimize the recovery of soybean and mustard allergens from wheat flour. The extracted proteins were analyzed using a bottom-up proteomics approach involving trypsin digestion, coupled with reversed-phase liquid chromatography and mass spectrometry in multiple reaction monitoring (MRM) mode. Two key allergenic proteins, Glycinin G1 and 11S Globulin, were selected as representative for soybean and mustard, respectively. The identified quantifier marker of Glycinin G1 was VLIVPQNFVVAAR (*m/z* 713.431^2+^), while FYLAGNQEQEFLK (*m/z* 793.896^2+^) and VFDGELQEGR (*m/z* 575.280^2+^) were designated as qualifier markers. The selection of specific marker peptides for mustard proved challenging due to the high structural similarity among proteins from *Sinapis alba* and other members of the *Brassicaceae* family. For 11S Globulin, FNTLETTLTR (*m/z* 598.319^2+^) was recognized as the quantifier marker, with VTSVNSYTLPILQYIR (*m/z* 934.019^2+^) serving as the qualifier marker. The developed method underwent thorough validation for linearity, limit of detection (LOD), limit of quantification (LOQ), recovery, repeatability, and reproducibility, as well as potential matrix and processing effects. This strategy successfully facilitated the identification and quantification of soybean and mustard allergenic proteins in complex, processed food matrices, including naturally contaminated flour and cookies. These findings enhance food safety monitoring and regulatory compliance, thereby helping to mitigate allergen-related risks in wheat-based products.

## 1. Introduction

Food allergens are predominantly proteins capable of triggering immune responses in sensitive individuals, ranging from mild symptoms like skin rashes to severe, life-threatening anaphylaxis [1,2,3]. Recognizing the growing public health impact, the World Health Organization (WHO) has classified food allergies as the fourth major global public health concern. Similarly, the European Union (EU), through Regulation EC/1169/2011, has identified 14 common allergenic ingredients [4], including milk, eggs, soy, mustard, lupin, molluscs, crustaceans, fish, tree nuts, cereals containing gluten, peanuts, celery, sesame, and sulfur dioxide, which must be clearly labeled on food packaging to protect allergic consumers [5]. However, with evolving dietary trends and the introduction of novel food sources such as insects and microalgae, this list is likely to expand. To ensure food safety, reliable analytical methods are crucial for detecting allergenic ingredients, particularly in processed foods, where heat, pressure, and pH variations may modify protein structures, potentially altering their detectability [6,7,8,9,10]. Traditional, direct and indirect immunochemical assays, such as enzyme-linked immunosorbent assay (ELISA) [11,12,13], and polymerase-chain-reaction (PCR)-based methods [14,15,16,17] are widely used for allergen detection due to their speed and simplicity. Examples include ELISA, radioallergosorbent test (RAST), enzyme-linked allergosorbent test (EAST), and combinations of gel electrophoresis with immunoblotting, all of which rely on antigen–antibody binding detection. Although some of these tests are rapid and straightforward, they have notable limitations, particularly the occurrence of false positives. These can result from interfering substances present in complex matrices or from sample treatments, such as heat processing, which may cause cross-reactivity phenomena. Among the indirect methods, the PCR stands out for its ability to detect DNA sequences that encode specific allergenic proteins. This technique involves several steps, including DNA extraction, purification, amplification, and detection. The PCR method is highly sensitive and avoids cross-reactivity, but it is relatively expensive and requires conversion factors to estimate the presence of allergenic proteins from the DNA level [10,18]. To overcome these limitations, liquid chromatography coupled with mass spectrometry (LC-MS) has emerged as a reliable alternative, allowing the direct identification of allergenic proteins through a bottom-up proteomics approach. This technique detects marker peptides unique to allergenic proteins, enabling the simultaneous quantification of multiple allergens in a single analysis, optimizing both time and costs [19,20,21,22,23,24]. In this study, we employ reversed-phase liquid chromatography (RPLC) coupled with electrospray ionization mass spectrometry (ESI-MS) to qualitatively and quantitatively determine soybean and mustard allergens in wheat flour and processed food products. Specific marker peptides were selected and categorized as either qualifiers or quantifiers for the soybean and mustard allergenic proteins. Subsequently, a targeted MS method was developed using a multiple reaction monitoring (MRM) approach to selectively detect these peptides. This strategy enhances the selectivity of the analytical method by enabling the detection of product ions with predefined *m/z* values that originate from a specific precursor *m/z*. As a result, it minimizes the detection of isobaric peptides and improves the accuracy of allergen identification.

Cross-contamination of wheat with mustard can occur due to agricultural practices, for example. One key contributor is green manuring, where mustard crops are rotated with wheat to exploit nutrient release and mustard’s natural pest control properties. Another contributing factor is crop rotation, where different plant species are cultivated sequentially on the same land to maintain soil health and reduce the need for chemical inputs. Additionally, the use of organic fertilizers derived from plant waste can also introduce traces of allergenic species into the soil. These sustainable practices help reduce dependence on synthetic fertilizers and pesticides, benefiting the environment both by minimizing the use of harmful compounds and by limiting their production. Certain plants used in these systems possess natural fungicidal and herbicidal properties, contributing to a more balanced and resilient agroecosystem. However, these methods may lead to unintended cross-contamination between different plant species and, even at trace amounts, can pose a serious risk to allergic individuals. Some of these seeds, such as soy, mustard, and lupin, are included in the list of 14 allergenic ingredients identified by the EU. Similarly, soybean contamination may occur during transportation and storage using shared infrastructure like silos, trucks, or shipping containers. If these facilities are not thoroughly cleaned between uses, residual soybean material can inadvertently mix with wheat.

Several studies have successfully employed mass spectrometry for soybean allergen detection in various food products. Both high- and low-resolution MS have been utilized, leveraging different acquisition strategies such as data-dependent acquisition (DDA) [25,26] and MRM [27,28,29]. In MS-based allergen detection, specific marker peptides are monitored within complex and processed food matrices for robust identification and quantification [30,31]. For instance, Huschek et al. [32] developed an LC-MS method for the simultaneous detection of soybean (Gly m 6), sesame (Ses i 6), and lupin (β-conglutin) allergens in wheat flour, cookies, and soft bread. The marker peptide VFDGELQEGR was used to quantify Gly m 6, while LSAEFGSLR and LNALKPDNR served as qualifier peptides. The limit of quantification (LOQ) for wheat flour and cookies was 10 ppm, while for soft bread, it was 20 ppm. Additional studies have demonstrated the detection of soybean allergens in diverse foodstuffs: Heick et al. [33] detected soy allergens in incurred samples with a limit of detection (LOD) of 24 ppm; Gu et al. [34] investigated chocolate matrices, reporting a LOQ between 1 and 4 ppm; Pilolli et al. [35] analyzed cookies, achieving a LOD of 6 ppm and a LOQ of 19 ppm; and Hoffmann et al. [36] detected soybean allergens in meat products with a LOD of 4 ppm.

Compared to soy, mustard allergen detection in complex and processed foods remain less explored. Posada-Ayala et al. [37] focused on Sin a 1 (2S albumin) mustard allergens in mustard sauce and salty biscuits using SRM-MS (selected reaction monitoring-MS), targeting peptides with a 100% match to the 2S seed storage protein family. This study highlighted the high structural similarity of mustard proteins within the *Brassicaceae* family, making selective detection more challenging. Furthermore, L’Hocine et al. [38] emphasized cross-reactivity between different mustard varieties, showing that immunoglobulin E (IgE) antibodies recognize shared epitopes across species. From a structural perspective, Hummel et al. [39] characterized isoforms of Sin a 1 using bottom-up, middle-down, and top-down proteomic approaches, providing insight into mustard’s allergenic protein diversity.

To address the risk of soy and mustard contamination in wheat-derived products, we propose a highly selective and sensitive RPLC-MS method capable of simultaneous identification and quantification of these allergens in processed wheat foodstuffs. Specifically, we evaluated different protein extraction protocols [30,40,41,42,43] to uncover the most effective method for recovering allergenic proteins from wheat flour. The analytical method was validated for key performance parameters, including linearity, LOD, LOQ, recovery, repeatability, reproducibility, and matrix effects. A bottom-up proteomic approach targeting quantifier marker peptides from Glycinin G1 (soy) and 11S Globulin (mustard) in naturally contaminated flour and cookies was applied. By establishing a robust and validated MS-based approach, this study aims to enhance food safety monitoring, ensuring compliance with regulatory requirements and minimizing allergen-related risks for consumers.

## 2. Results and Discussion

### 2.1. Evaluation of Protein Extraction Protocols for Allergen Identification

**Figure 1:**
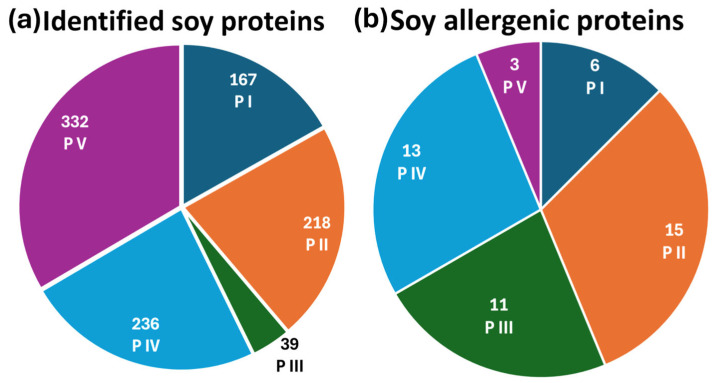
Pie charts of (**a**) the total number of identified soy proteins and (**b**) the identified soy allergenic proteins across all five extraction protocols.

To determine the most effective extraction protocol for protein and allergen identification, soy and mustard extracts were analyzed using reversed-phase liquid chromatography coupled with electrospray ionization Fourier-transform mass spectrometry in data-dependent acquisition mode (RPLC-ESI(+)-FTMS/MS DDA). The total ion current (TIC) chromatograms of tryptic digests from five extraction protocols applied to soy flour are presented in Figure S1, with plots A–E corresponding to protocols P I, P II, P III, P IV, and P V, respectively. Protein and allergen identification was performed by processing the raw data through ProteomeDiscoverer™ (PD) software (version 2.4), utilizing the *Glycine max* and *Sinapis alba* databases for soy and mustard samples, respectively. PD allowed us to recognize the amino acid sequences of tryptic peptides based on tandem mass spectra acquired in DDA mode, associating them with corresponding proteins. This approach generated a comprehensive list of detected proteins along with their sequence coverage. For soy samples, as depicted in Figure 1a, the number of proteins identified with coverage percentages exceeding 10% varied among protocols: 167 proteins were identified using protocol P I, 218 with P II, 39 with P III, 236 with P IV, and 332 with P V. The full list of identified proteins across all extraction methods is detailed in Table S1.

At first glance, protocol P V appeared to provide the most comprehensive identification of the soybean proteome, detecting the highest number of proteins. However, a detailed analysis focusing specifically on allergenic proteins revealed a different trend. As shown in Figure 1b, the number of allergenic proteins identified by the five extraction protocols was as follows: 6 for P I, 15 for P II, 11 for P III, 13 for P IV, and 3 for P V. The list of identified soy allergenic proteins, along with their sequence coverage percentages, is reported in Table 1. These findings indicate that protocol P II was the most effective in extracting allergenic proteins. A further comparison of protocols P II and P V, which yielded the best results in terms of allergen identification and total protein recognition, respectively, was carried out. To deepen the analysis, the Grand Average of Hydropathy (GRAVY) index was calculated for each allergenic protein and its associated peptides. GRAVY assesses the overall hydropathy of a protein or peptide by averaging the hydropathy indices of its constituent amino acids. Positive and negative values, respectively, reflect hydrophobic and hydrophilic properties. For soybean allergenic proteins, GRAVY values ranged from −1.07 to +0.13, with 16 proteins exhibiting negative values, one showing a GRAVY value of zero, and one allergen displaying a positive value (+0.13). These findings suggest that soybean allergens are predominantly hydrophilic, explaining their enhanced solubility in the aqueous medium used in protocol P II. For example, considering the allergenic protein P19594, 11 peptides (42% coverage) were identified using P II, compared to only 2 peptides (15% coverage) detected with P V. Notably, eight peptides from P II and one from P V exhibited negative GRAVY values, indicating their hydrophilic nature. This suggests that the tryptic peptides generated by P II displayed higher chromatographic intensity, facilitating fragmentation and identification in DDA mode. Among the soybean allergens, Glycinin G1 (UniProt accession code P04776), known as Gly m 6, was selected as the representative protein. The Gly m 6 family (Glycinin, 11S Globulin, Legumin) belongs to the storage protein category and represents the most abundant soybean storage protein, accounting for 40–50% of the total seed protein, sometimes exceeding 50%. Gly m 6 allergens are classified as Class I food allergens, capable of inducing food allergies through primary sensitization and potentially triggering severe reactions, including anaphylaxis, in sensitized individuals [44]. Furthermore, Gly m 6 contains linear epitopes structured within the protein folds, with in vitro tests confirming IgE binding between Gly m 6 and sera from soybean-sensitized individuals [45]. Structural analyses have also been conducted to explore variations influencing physicochemical, functional, and physiological properties [46,47,48].

Based on the results obtained for soy allergens, the GRAVY values of mustard allergenic proteins, as recognized by WHO/IUIS (International Union of Immunological Societies), were calculated to determine the most appropriate extraction protocol. Notably, all four known mustard-allergenic proteins displayed negative GRAVY values. Given the findings for soybean flour, protocols P II, P III, and P IV were chosen for extracting mustard-allergenic proteins. The TIC chromatograms corresponding to these protocols are shown in Figure S2, plots a–c, respectively. The chromatographic runs in DDA mode were processed using PD software, and the identified proteins are listed in Table S2. Specifically, 117 proteins were identified using protocol P II, 6 proteins with P III, and 75 proteins with P IV. It should be noted that the lower number of identified proteins in mustard compared to soybean flour can be attributed to the significantly smaller size of the *Sinapis alba* database (273 entries) compared to the *Glycine max* database (>123,000 entries). The allergenic proteins identified through the three protocols are listed in Table 2. Among the detected allergenic proteins, 11S Globulin (UniProt accession code Q2TLW0), recognized as allergen Sin a 2, was selected as the representative mustard protein. WHO/IUIS recognizes four major mustard allergens: Sin a 1, a seed storage protein from the 2S albumin family; Sin a 2, part of the 11S Globulin family; Sin a 3, a non-specific lipid transfer protein (nsLTP); and Sin a 4, identified as profilin [38]. Among these, the 2S albumin and 11S Globulin families serve as the primary storage proteins in mustard and are considered the most significant allergens for this ingredient [49].

### 2.2. Identification of Marker Peptides of Soy and Mustard Allergenic Proteins

The identification of Glycinin G1 and 11S Globulin proteins was achieved using (PD) software, revealing a coverage of 74% for Glycinin G1 and 50% for 11S Globulin. Figure S3 illustrates the extracted ion current (EIC) chromatograms of four distinct peptides from these allergenic proteins, with normalized intensities. In the case of Glycinin G1 (Figure S3a), the selected peptides, in order of elution, were QIAKNLQGENEGEDR (*m/z* 836.910^2+^), RFYLAGNQEQEFLK (*m/z* 871.947^2+^), VLIVPQNFVVAAR (*m/z* 713.431^2+^), and NAMFVPHYNLNANSIIYALNGR (*m/z* 1246.613^2+^). When the intensity of the most abundant peptide (*m/z* 713.431^2+^) was set as the reference (unitary value), the relative intensities of the other three peptides were 3%, 40%, and 2.5%, respectively. This variation in peptide intensities suggests differences in the proteolytic enzyme digestion and ionization efficiency in the ESI source. Remarkably, the peptide at *m/z* 713.431^2+^ exhibited a highly intense signal, making it a strong candidate as a marker peptide. For 11S Globulin (Figure S3b), the selected peptides were FNTLETTLTR (*m/z* 598.319^2+^), TNANAMISTLAGR (*m/z* 660.340^2+^), GILQGSAMVLPK (*m/z* 607.351^2+^), and DACNLDNLDVLQPTEVIK (*m/z* 1029.012^2+^). When normalizing the most intense peptide (*m/z* 1029.012^2+^) as unitary, the relative intensities of the remaining peptides were determined as 70%, 25%, and 33%, respectively.

To identify marker peptides for Glycinin G1 (soy) and 11S Globulin (mustard), in silico digestion was performed using the PeptideMass tool (ExPASy) with trypsin as the proteolytic enzyme and no missed cleavages. Candidate peptides were filtered based on established literature criteria [30,31], ensuring optimal length (7–20 amino acids), absence of cleavage site errors, and exclusion of amino acids prone to modifications such as methionine oxidation, cysteine disulfide formation, and asparagine-glycine (NG) sequence motif deamidation. In this context, peptides are typically selected within a length range of 7 to 20 amino acids because shorter sequences may not be specific enough to uniquely identify the target allergenic protein, while longer ones often show reduced ionization efficiency during MS analysis. This range is considered a good balance between specificity, ionization performance, and reproducibility, while peptides resulting from incomplete enzymatic digestion, known as missed cleavages, are avoided, as well as those containing amino acids that are particularly susceptible to chemical modifications, since these factors can introduce variability and compromise the consistency of the analytical workflow [31].

A total of 10 marker peptides were identified for Glycinin G1 (ranging from 9 to 13 amino acids), while 12 marker peptides were found for 11S Globulin (ranging from 9 to 19 amino acids). The presence and uniqueness of each peptide was verified using BLAST (blastp suite) against the UniProtKB/Swiss-Prot and non-redundant protein sequences (nr) databases. This analysis confirmed 10 soy-specific marker peptides via UniProtKB/Swiss-Prot and five via the non-redundant protein sequences database (Table 3). A doubly charged peptide at *m/z* 713.431^2+^ was also detected in legumin A-like protein from *Lotus japonicus*, a plant in the same subfamily as *Glycine max*. Despite this, it was chosen as a quantifier peptide due to its high intensity, favorable signal-to-noise (S/N) ratio, and thermal stability, supported by previous studies [31,34,35]. Conversely, peptides FLVPPQESQ and LSAEFGSLR (see Table 3) were identified in bacterial species (*Pseudomonadota bacterium* and *Cyanobacterium*) and were therefore excluded.

For mustard, marker peptide selection was more complex due to high protein sequence similarity between *Sinapis alba* and other *Brassicaceae* species (e.g., *Arabidopsis thaliana*, *Brassica napus*, *Brassica rapa*). This made it challenging to define species-specific peptides. Table 3 indicates that only four peptides could serve as potential markers when analyzed using the non-redundant protein sequences database, and just one (QSLGVPPQVK) was identified using the UniProtKB/Swiss-Prot database. However, the intensity and thermal stability of these peptides were insufficient for analytical use. Nevertheless, the presence of *Sinapis alba* in wheat samples was confirmed, as the wheat was cultivated using green manuring practices. Based on the findings in Table 3, the following peptides were selected as marker peptides: for Glycinin G1, VLIVPQNFVVAAR (*m/z* 713.431^2+^) was chosen as the quantifier marker, while FYLAGNQEQEFLK (*m/z* 793.896^2+^) and VFDGELQEGR (*m/z* 575.280^2+^) were selected as qualifier markers. For 11S Globulin, FNTLETTLTR (*m/z* 598.319^2+^) was identified as the quantifier marker, with VTSVNSYTLPILQYIR (*m/z* 934.019^2+^) serving as the qualifier marker.

**Figure 2:**
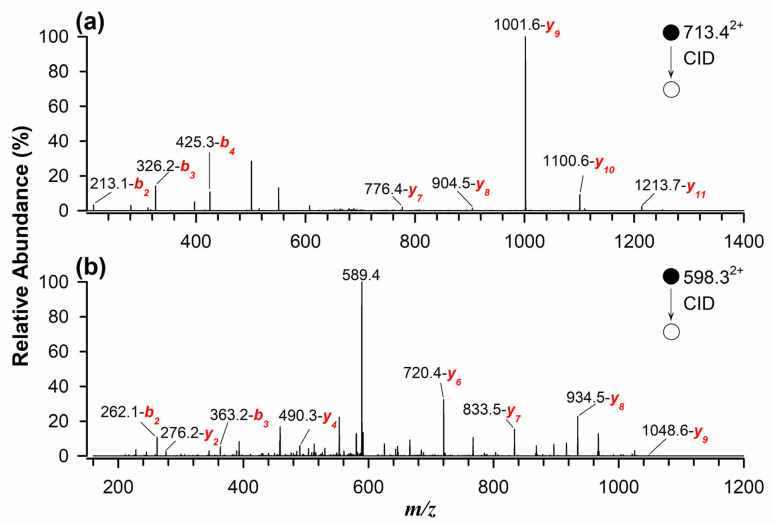
Tandem MS spectra using RPLC-ESI(+)-CID-MS/MS for doubly protonated marker peptides as quantifiers of (**a**) Glycinin G1 at *m/z* 713.4^2+^ and (**b**) 11S Globulin at *m/z* 598.3^2+^.

The identification and quantification of marker peptides in processed samples proved challenging due to potential matrix interferences. When naturally contaminated flour samples were analyzed using RPLC-ESI(+)-FTMS, multiple interferences were observed. To overcome this issue, a targeted approach using MRM was implemented for the quantitative determination of allergenic proteins in standard solutions, spiked and incurred samples, and naturally contaminated samples. This method enhances selectivity and sensitivity by monitoring the most intense fragment ions of marker peptides during tandem mass spectrometry analysis [29,33]. As illustrated in Figure 2a, the MS/MS spectrum, obtained in collisional induced dissociation (CID) fragmentation mode, of the quantifier peptide for Glycinin G1 (*m/z* 713.4^2+^) identified the most intense product ions at *m/z* 1001.6 (*y_9_*), 1100.6 (*y_10_*), and 1213.7 (*y_11_*). Similarly, Figure 2b presents the MS/MS spectrum of the quantifier peptide for 11S Globulin (*m/z* 598.3^2+^), with dominant product ions at *m/z* 720.4 (*y_6_*), 833.5 (*y_7_*), and 934.5 (*y_8_*). This targeted approach significantly improved peptide detection, even at low concentrations in complex and processed food matrices.

### 2.3. Analytical Method Validation for Glycinin G1 and 11S Globulin Quantification

The validation of the proposed analytical method was conducted by analyzing standard solutions, spiked and incurred samples, and naturally contaminated samples under identical experimental conditions using MRM mode. Initially, standard solutions obtained through enzymatic digestion of protein extracts from soy and mustard flours, following protocol II, were analyzed. The matrix effect for naturally contaminated flour samples was evaluated by using allergen-free flour spiked with soy and mustard flour before protein extraction, as described in Section 2.4. Calibration curves were constructed by plotting the chromatographic peak area of each quantifier marker peptide against the concentration of the allergenic ingredient in mg of ingredient per kg of the matrix (mg_ingredient_/kg_matrix_). These curves exhibited excellent linearity for both marker peptides, with an R ^2^ value of 0.999 in each case, as detailed in Table 4.

The matrix effects, calculated at 73.59 ± 0.02% for soy and 86.68 ± 0.06% for mustard, indicated minimal interference from other flour components, thereby ensuring analytical accuracy. The LOD and LOQ were expressed in mg of total protein per kg of the matrix (mg_Prot.Tot_/kg_matrix_), based on the protein content of 40% for soy flour and 26% for mustard flour, as indicated on the respective product labels. The LOD and LOQ for soy were determined to be 2.8 and 9.2 mg_Prot.Tot_/kg_matrix_, respectively, while for mustard, these values were 2.9 and 9.8 mg_Prot.Tot_/kg_matrix_. The LOD and LOQ values obtained for soybean allergenic proteins in unprocessed samples align with those reported by Huschek et al. [32]. However, for mustard allergens, no direct comparison with literature data was possible due to the absence of studies reporting the identification and quantification of mustard-allergenic ingredients in complex matrices using LC-MS approaches.

Recovery was assessed at two concentration levels by comparing the peak areas of quantifier marker peptides for the Glycinin G1 and 11S Globulin allergenic proteins in spiked flour and cookies with those in fortified samples. For flour samples, the recovery percentage ranged from 48 ± 3 to 73 ± 5 for soybean, at lower and higher concentrations, respectively, and from 88 ± 7 to 93 ± 7 for mustard.

For processed food matrices such as cookies, both the matrix effect on allergen quantification and the impact of thermal processing were evaluated. In general, food processing methods, such as heating, high-pressure treatment, and irradiation, can induce protein denaturation or modification, leading to alterations in the protein profile [50,51,52] and potentially causing an underestimation of allergenic protein content, posing risks to sensitized individuals [53]. Moreover, during food processing, additional phenomena may occur, such as the formation of protein aggregates or structural modifications like, for example, glycation in the Maillard reaction [54,55]. These changes can affect the molecular weight of the protein and, consequently, the peptides generated during enzymatic digestion. As a result, the *m/z* ratios of the selected marker peptides used for method validation may shift, potentially compromising the accuracy of allergen detection and quantification. To address these effects, both spiked and incurred cookie samples were analyzed. Spiked samples involved the addition of allergenic ingredients to blank cookies before protein extraction, whereas incurred samples included allergenic ingredients incorporated into the dough before baking. Calibration curves were generated for both sample types, and the parameters are reported in Table 4. The matrix effect for cookies was calculated at 75.51 ± 0.02% for soy and 83.27 ± 0.06% for mustard, values comparable to those observed for wheat flour. Thermal processing significantly impacted soy allergen quantification, with a processing effect of 31.60 ± 0.03%, indicating a substantial reduction in the analytical signal for the marker peptide due to baking. Failure to consider this effect could result in severe underestimation of soy allergen content in processed matrices. In contrast, mustard allergens exhibited greater thermal stability, with a processing effect of 76.64 ± 0.06%, suggesting enhanced resilience of the marker peptide under thermal conditions. The combined matrix and processing effects were determined to be 23.86 ± 0.03% for soy and 63.83 ± 0.03% for mustard. Based on the calibration curves for incurred samples, the following LOD and LOQ values were established for the allergenic ingredients: 6.7 and 22.2 mg_Prot.Tot_/kg_matrix_ for soy, and 4.3 and 14.2 mg_Prot.Tot_/kg_matrix_ for mustard. Additionally, for cookie samples, the recovery percentage ranged from 40 ± 2 to 75 ± 5 for soybean and from 79 ± 7 to 95 ± 9 for mustard. Analytical repeatability and reproducibility, including extraction and digestion steps, were evaluated by analyzing three independent extracts of cookies and flour samples spiked at 400 mg_ingredient_/kg_matrix_, with each sample analyzed in triplicate over three consecutive days. Repeatability ranged from 2.8% to 5.6% for soybeans and from 1.4% to 3.1% for mustard, while reproducibility was determined to be 16% for both allergens.

These findings underscore the robustness and reliability of the analytical method for detecting allergenic proteins in both raw and processed food matrices, ensuring consumer safety. In the case of soybean allergens in processed matrices, the LOD and LOQ values align with those reported in the literature for similar samples, such as bread [33] and cookies [35]. However, as previously mentioned, no comparable studies for mustard allergens are available for reference.

### 2.4. Quantification of Soy and Mustard Allergens in Naturally Contaminated Samples

The quantification of allergenic proteins in naturally contaminated wheat processed samples was conducted using the previously established analytical method in the MRM mode. Specifically, wheat flour naturally contaminated with soy and mustard allergenic ingredients, due to the practice of green manuring during wheat cultivation, was analyzed using RPLC-ESI(+)-MS in the MRM mode following protein extraction with Protocol II and enzymatic digestion with trypsin.

**Figure 3:**
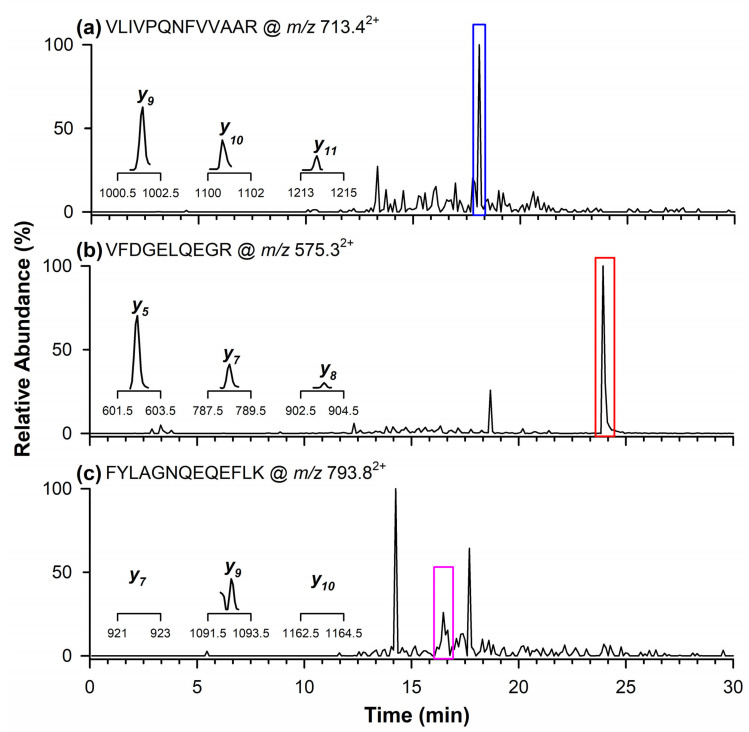
Chromatograms obtained through RPLC-ESI-LIT-MS/MS in the MRM mode illustrating the occurrence of quantifier marker peptide (**a**) of Glycinin G1 at *m/z* 713.4^2+^, and qualifier marker peptides at (**b**) *m/z* 575.3^2+^ and (**c**) 793.8^2+^. In the inserts are the single product ions monitored in the MRM mode for each marker peptide.

The presence of the quantifier marker peptide related to the Glycinin G1 protein from soy was detected at a retention time of 18.1 min (Figure 3a) with product ions corresponding to *m/z* values of *y_9_*, *y_10_*, and *y_11_*. Figure 3b,c display the chromatographic peaks of the qualifier peptides for Glycinin G1, observed at retention times of 23.9 min (Figure 3b) and 16.5 min (Figure 3c). Specifically, the inset in Figure 3b highlights the product ions at *m/z* 602.3 (*y_5_*), 788.4 (*y_7_*), and 903.4 (*y_8_*), whereas in Figure 3c, only the product ion at *m/z* 1092.5 (*y_9_*) is reported, as the other two ions at *m/z* 921.5 (*y_7_*) and 1163.6 (*y_10_*) were not detectable in the naturally contaminated wheat flour.

**Figure 4:**
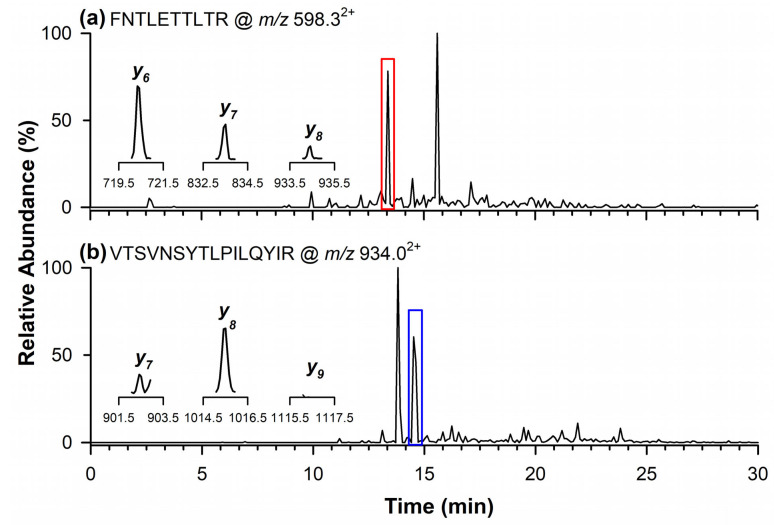
Chromatograms by RPLC-ESI-LIT-MS/MS in the MRM mode of quantifier marker peptide (**a**) of 11S Globulin at *m/z* 598.3^2+^, and qualifier marker peptide at (**b**) *m/z* 934.0^2+^. In the inserts are the single product ions monitored in the MRM mode for each marker peptide.

Figure 4a shows the chromatographic peak of the quantifier marker peptide for 11S Globulin at *m/z* 598.3^2+^, detected at a retention time of 13.4 min. The inset displays the signals of the product ions monitored in the MRM mode at *m/z* 720.4 (*y_6_*), 833.5 (*y_7_*), and 934.5 (*y_8_*). In Figure 4b, the chromatographic peak at 14.5 min corresponds to the qualifier marker peptide at *m/z* 934.0^2+^. In this case, the inset highlights the product ions at *m/z* 902.6 (*y_7_*) and 1015.6 (*y_8_*), while the ion at *m/z* 1116.7 (*y_9_*) was not detectable in the analyzed sample.

The concentrations of these allergenic proteins were determined based on the chromatographic peak areas, utilizing the calibration curve parameters obtained from wheat flour samples spiked with soy and mustard, while also accounting for the previously described matrix effect. The results indicated concentrations of 26.5 ± 1.1 mg_Prot.Tot_/kg_matrix_ for soy, assuming a 40% protein content, and 10.5 ± 1.2 mg_Prot.Tot_/kg_matrix_ for mustard, based on a 26% protein content. The validity of the developed analytical method was further evaluated for its applicability in identifying and quantifying these allergenic proteins in a processed sample, specifically cookies prepared in the laboratory using naturally contaminated flour. The analysis of tryptic digests from protein extracts, following Protocol II, successfully enabled the detection of both allergenic proteins in the processed sample. This identification was achieved using the chromatographic peaks of both quantifier and qualifier marker peptides.

The concentration of the soy allergenic ingredient was determined through the chromatographic peak area of the quantifier marker peptide at *m/z* 713.4^2+^ in the MRM mode, as illustrated in Figure S4. The concentration calculation was performed by applying the calibration curve parameters derived from incurred cookie samples, accounting for both processing and matrix effects. The total protein concentration was found to be 26 ± 9 mg_Prot.Tot_/kg_matrix_. In contrast, the quantification of the mustard allergenic ingredient in the naturally processed contaminated sample was hindered by significant background noise. The chromatographic peak corresponding to the quantifier and qualifier marker peptides exhibited a very low signal, resulting in a concentration value below the LOQ established from the calibration curve of incurred samples.

Further investigation is needed to better understand the behavior of mustard allergens in processed samples derived from contaminated wheat flour, particularly the disappearance of the quantifier marker peptide. This effect may be influenced by the higher processing temperatures used in cookie production, combined with the low concentration of this allergenic ingredient in the examined wheat sample. Additionally, the baking temperature for cookies is significantly higher than the relatively lower temperatures applied during pasta drying. From this perspective, it may be possible to detect the quantifier marker peptide of mustard allergenic protein in pasta. Alternatively, other factors may also contribute to this phenomenon, necessitating a more detailed analysis.

## 3. Material and Methods

### 3.1. Chemicals

Water, hexane, methanol, acetonitrile, acetone, chloroform, formic acid, trifluoroacetic acid, Tris (hydroxymethyl)aminomethane hydrochloride (Tris-HCl), DL-Dithiothreitol (DTT), iodoacetamide (IAA), ammonium bicarbonate (ABC), porcine pancreas trypsin, ethylenediaminetetraacetic acid (EDTA), 2-chloroacetamide (CAA), 2-Amino-2-hydroxymethyl-propane-1,3-diol (TRIZMA), sodium tetraborate, vitamin C, polyvinylpolypyrrolidone (PVPP), triton X-100, β-mercaptoethanol, sucrose, ammonium sulfate, trizma, ammonium hydroxide, sodium hydroxide, tris (2-carboxyethyl)phosphine (TCEP), sodium deoxycholate (SDC), and phenol (equilibrated with 10 mM Tris-HCl, pH 8.0, 1 mM EDTA) were purchased from Merck (Milan, Italy). All solvents used were LC-MS-grade, except for hexane and chloroform (HPLC-grade). The RapiGest surfactant was bought from Waters Corporation (Milan, Italy). Wheat and flour were provided by the Food Safety Lab S.r.l. (Corato, Italy) after performing a screening with ELISA. Soy and mustard flours were purchased from local supermarkets.

### 3.2. Protein Extraction Protocols

Five preliminary protocols were assessed for the extraction of allergenic proteins from soy and mustard flours, which were used as standard reference materials.

Protocol I [43]: A volume of 5 mL of buffer (i.e., 10 mM TCEP, 40 mM CAA, 100 mM ABC, and 1% SDC, pH 8.5) was added to 0.5 g of sample, followed by shaking. The samples were then sonicated on ice for five ON/OFF cycles of 20 s each, under the following conditions: 60% amplitude, 65% power, 80% pulse cycle, and 30 Wh energy output. After sonication, the mixture was shaken at 400 rpm at 80 °C for 10 min, followed by centrifugation at 5000× *g* for 15 min. The supernatant (100 µL) was then dried under nitrogen (N_2_) for protein digestion.

Protocol II [42]: A volume of 5 mL of hexane was added to 0.5 g of sample, and the mixture was stirred at 25 °C and 400 rpm for 15 min, then centrifuged at 5000× *g* for 5 min. The supernatant was discarded, and the lipid removal step was repeated twice. To facilitate slurry formation, 800 µL of water was added, and the mixture was kept at 20 °C for two hours, with intermittent stirring every 15 min for 30 s. Subsequently, 5 mL of water was added, and sonication on ice was performed under the same conditions as previously described, with eight ON/OFF cycles of 30 s each. The pH was measured and adjusted using 2N NaOH solution, followed by centrifugation at 5000× *g* for 60 min. Finally, 100 µL of the interphase was dried under N_2_ for protein digestion.

Protocol III [40]: A volume of 5 mL of buffer (i.e., 100 mM EDTA, 100 mM Trizma, 50 mM sodium tetraborate, 50 mM vitamin C, 1% (*w*/*v*) PVPP, 1% (*v*/*v*) Triton X-100, 2% (*v*/*v*) β-mercaptoethanol, and 30% (*w*/*v*) sucrose) was added to 0.5 g of sample and shaken at 800 rpm and 25 °C for 30 min. The samples were then sonicated on ice under the same conditions as previously described for five ON/OFF cycles of 20 s each, followed by centrifugation at 15,000 rpm at 4 °C for 20 min. The supernatant (1 mL) was then mixed with 2 mL of phenol solution and stirred at 800 rpm for 10 min, followed by centrifugation at 15,000 rpm at 4 °C for 20 min. An equal volume of buffer was added to the supernatant and stirred at 800 rpm and 25 °C for 10 min. The mixture was centrifuged again under the same conditions, and the supernatant was treated with 5 mL of methanol saturated with ammonium sulfate before being stored overnight at −20 °C. The precipitated pellet was recovered after centrifugation at 15,000 rpm at 4 °C for 20 min, resuspended in 1 mL of chilled methanol, stirred, and centrifuged. The pellet was washed with chilled acetone, then dried under N_2_ and subjected to protein digestion.

Protocol IV [41]: A volume of 1 mL of 1.25% (*v*/*v*) TFA was added to 0.15 g of the sample, followed by sonication on ice for five ON/OFF cycles of 30 s each [56]. After centrifugation at 15,000 rpm for 15 min, the supernatant was collected and neutralized, and 100 µL was dried under N_2_ for protein digestion.

Protocol V [30]: A volume of 5 mL of 50 mM Tris-HCl was added to 0.5 g of sample, followed by sonication on ice under previously described conditions, with five ON/OFF cycles of 30 s each. The samples were then incubated at 60 °C and 800 rpm for 2 h, centrifuged at 5000× *g* for 30 min, and 100 µL of the supernatant was collected and dried under N_2_ for protein digestion.

### 3.3. Enzymatic Digestion Procedure

The enzymatic digestion phase was conducted by adding 50 µL of Rapigest (1 mg/mL) and 50 µL of ABC buffer (50 mM) to the dried protein extracts for solubilization. The mixture was then treated with 10 µL of DTT (50 mM) and stirred at 60 °C and 400 rpm for 30 min. Following this, 10 µL of IAA (150 mM) was introduced, and the samples were incubated in the dark at 25 °C and 400 rpm for 30 min. Subsequently, 10 µL of trypsin (1 µg/µL in 25 mM ABC buffer) was added, and the samples were stirred overnight at 37 °C and 400 rpm to complete protein digestion. To terminate tryptic digestion, 5 µL of formic acid (98% *w*/*v*) was added. This digestion procedure was applied to all extracts obtained from the various extraction protocols, except for those prepared using protocol I buffer, as this buffer already contained reducing and alkylating agents. For these specific extracts, enzymatic digestion was performed by directly adding 10 µL of trypsin (1 µg/µL in 25 mM ABC buffer) [57].

### 3.4. Preparation of Standard Solutions and Sample Analysis

Standard calibration solutions were prepared using soy and mustard flour, along with spiked wheat flour, spiked cookies, and incurred cookies. Specifically, standard solutions were prepared at 0.2–4 μg_ingredient_, starting from 0.1 g of soy/mustard flours extracted using Protocol II, with a modified extraction volume. For all other samples, protein extraction was carried out using Protocol II, starting from 0.5 g of sample. To evaluate matrix effects, two different matrices were employed: wheat flour and cookies. In detail, 10 mg of each allergenic ingredient was added to 0.49 g of sample, followed by protein extraction and enzymatic digestion. Serial dilutions were prepared using wheat flour or cookie extracts devoid of allergenic ingredients, achieving a concentration range of 20–400 mg_ingredient_/kg_matrix_ across five levels (400, 200, 100, 40, and 20 mg_ingredient_/kg_matrix_).

The processing effect was evaluated using incurred cookies prepared in the laboratory, following the composition: 50 g of flour, 22.5 g of sugar, 0.125 g of NaCl, 0.125 g of ABC, 11.25 g of olive oil, 20 g of water, and 1.7 g of soy and mustard flour. These cookies were baked at 180 °C for 20 min, resulting in a final weight of 82 g due to water loss. After protein extraction and enzymatic digestion, calibration curves for incurred cookies were constructed using concentration levels within the range of 20–400 mg_ingredient_/kg_matrix_. Matrix effects were assessed as the percentage ratio between the slopes of calibration curves for spiked samples (wheat flour or cookies) and standard solutions, while the processing effect was determined as the ratio between the calibration slope of incurred and spiked cookies. The combined effect, accounting for both processing and matrix effects, was evaluated as the percentage ratio between the slope of the calibration curve for incurred cookies and the slope of standard solutions. Additionally, LOD and LOQ were calculated for both spiked and incurred samples, using values of three and ten times, respectively, the ratio between the intercept standard deviation and the slope of calibration curves.

Recovery was assessed at two concentration levels (20 and 400 mg_ingredient_/kg_matrix_) by comparing the peak area of quantifier marker peptides from Glycinin G1 and 11S Globulin in spiked cookies and flours with those in fortified matrices (2 and 40 µg_ingredient_/mL). Specifically, fortified matrices were prepared by adding soy and mustard standard solutions to blank matrix extracts, while spiked matrices were obtained by incorporating soy and mustard flours into the matrices before protein extraction.

Finally, analytical repeatability and reproducibility were evaluated by analyzing three independent extracts of cookies and flours, preliminarily spiked at 400 mg_ingredient_/kg_matrix_. Each sample was injected three times per day over three consecutive working days to assess consistency and reliability.

### 3.5. LC-MS(/MS) Experimental Conditions

LC-MS(/MS) analyses of tryptic digests were performed using a Dionex UltiMate 3000 HPLC system (Thermo Scientific, Waltham, MA, USA) coupled with a high-resolution/accuracy Q Exactive mass spectrometer (Thermo Scientific, MA, USA) in DDA mode for protein identification, and a low-resolution VelosPro mass spectrometer (Thermo Scientific) for quantitative analyses in multiple reaction monitoring (MRM) mode. Both systems employed an ESI interface operating in positive polarity. Chromatographic separation was carried out using a Phenomenex Aeris WIDWPORE 200 Å C18 column (250 × 2.1 mm, 3.6 µm) with a Phenomenex AJO 8783 WIDEPORE C18 guard column (2 × 2.1 mm ID) at a temperature of 40 °C. The injection volume was 5 µL, and the mobile phase composition was based on water (phase A) and acetonitrile (phase B), both containing 0.1% (*v*/*v*) of formic acid.

The gradient program for chromatographic runs was as follows: 0–2 min at 5% solvent B; 2–30 min linear from 5% to 60% (*v*/*v*) of B; 30–32 min linear from 60% to 100% B; 32–37 min isocratic at 100% of B; 37–40 min back to the initial composition, followed by 5 min equilibration time. High-resolution/accuracy mass spectrometry analyses were carried out in full scan and data-dependent mode in positive polarity. The ESI and ion optic parameters adopted for data-dependent analyses were the following: sheath gas flow rate, 60 (arbitrary units); auxiliary gas flow rate, 15 (arbitrary units); spray voltage, 4.0 kV in positive polarity; capillary temperature, 275 °C; S-lens radio frequency level, 100 arbitrary units. Positive MS full-scan spectra were acquired in the *m/z* range 400–1500 with 70 k of resolution using an automatic gain control (AGC) target of 3e6 and an injection time of 100 ms. The Full-MS/ddMS^2^ analyses on the top 8 ions experiments were performed using NCE fixed at 30 with a 17.5 k resolution, AGC of 3e6, IT fill time of 50 ms, isolation window of 2 *m/z*, minimum AGC of 5e3, and automatic dynamic exclusion, on bi- and tri-charged ions (excluding singly charged ions and ions with charges from +4 to +8). The control of the LC-MS instrumentation and the first processing of data were performed by the Xcalibur software 2.2 SP1.48 (Thermo Scientific), while mass spectrum processing was completed using SigmaPlot 14.5. The raw files in Full MS/ddMS^2^ mode ssed using ProteomeDiscoverer ^TM^ (version 2.4, Thermo Scientific) to identify proteins/allergens in standard solution samples extracted by different protocols previously reported, by employing the *Glycine max* and *Sinapis alba* database downloaded from Uniprot (https://www.uniprot.org/ accessed on 7 March 2025). The processing and consensus workflows for PD investigation were PWF_QE_Basic_SequestHT.pdProcessingWF and CWF_Basic.pdConsensusWF, respectively. Specifically, the parameters were the following: trypsin as the enzyme, 2 missed cleavages, minimum and maximum length of peptides equal to 6 and 144 amino acids, respectively, 10 ppm and 0.02 Da as tolerance for precursor and fragment ions, respectively, met-oxidation, acetyl, met-loss, met-loss + acetyl as dynamicmodifications, and carbamidomethylation of cysteines as a static modification.

Additionally, marker peptides for the two allergenic proteins selected as representative of soy and mustard ingredients, named Glycinin G1 and 11S Globulin, respectively, were identified through a two-step approach. First, an insilico digestion was performed using the PeptideMass tool (ExPASy) (https://web.expasy.org/peptide_mass/). Subsequently, based on established literature criteria [30,31], the selected peptides were analyzed using BLAST tools (blastp suite) to ensure specificity against all species.

## 4. Conclusions

This study evaluated several protein extraction protocols with varying buffer compositions to determine the most effective approach for identifying allergenic proteins from soybean and mustard in complex food matrices, such as wheat flour cross-contaminated with these allergens. Among the tested methods, the protocol employing water as the extraction solvent emerged as the most effective and safest for both ingredients. It enabled the identification of the highest number of allergenic proteins, providing superior coverage percentages compared to other extraction techniques. Two key allergenic proteins, namely Glycinin G1 for soybean and 11S Globulin for mustard, were selected as representative markers, and their quantifier and qualifier marker peptides were defined. To the best of our knowledge, the proposed RPLC-ESI-MS in multiple-reaction monitoring mode is the first method capable of simultaneously detecting and quantifying both soybean and mustard allergens, offering a powerful tool for food safety and allergen monitoring in processed wheat-derived foodstuffs.

## Figures and Tables

**Table 1:** List of identified soybean allergenic proteins utilizing five distinct protein extraction protocols. Each protocol is accompanied by its respective coverage percentage.

Protein Accession No.	Protein Coverage (%) for Each Extraction Protocol				
	P I ^a^	P II ^b^	P III ^c^	P IV ^d^	P V ^e^
P04776	4	74	60	67	--
P02858	7	68	53	44	--
P04405	--	67	53	59	--
P11828	--	64	43	49	--
O22121	17	59	--	--	--
F7J077	--	56	49	41	--
P11827	5	52	28	38	4
C6T588	13	45	--	13	--
P19594	--	42	30	41	15
P05046	--	39	16	20	--
C6T1Q7	--	33	16	41	10
O64458	--	29	7	4	--
Q9AVK8	--	29	--	--	--
C6K8D1	--	14	--	16	--
B1ACD5	--	9	--	--	--
F8WQS0	--	--	49	38	--
P04347	7	--	--	--	--

^a^ Buffer based on tris(2-carboxyethyl)phosphine, 2-chloroacetamide, ammonium bicarbonate, and sodium deoxycholate. ^b^ Water, subsequently neutralized with 2N NaOH. ^c^ Buffer based on EDTA, 2-amino-2-(hydroxymethyl)-1,3-propanediol (TRIZMA), sodium tetraborate, vitamin C, polyvinylpolypyrrolidone, Triton X-100, β-mercaptoethanol, and sucrose. ^d^ TFA 1.25% (*v*/*v*). ^e^ Tris-HCl.

**Table 2:** List of identified mustard allergenic proteins utilizing three protein extraction protocols: II, III, and IV. Each protocol is accompanied by its respective coverage percentage.

Protein Accession No.	Protein Coverage (%) for Each Extraction Protocol		
	P II	P III	P IV
P15322	39	37	13
Q2TLW0	50	54	--
Q2TLV9	39	43	--
E6Y2L9	89	17	--
E6Y2M0	10	--	10

**Table 3:** Putative marker peptides of Glycinin G1 (soybean) and 11S Globulin (mustard) for allergenic ingredients in wheat-derived foodstuffs.

Marker Peptide	BLAST (UniProtKB/Swiss-Prot)	BLAST (Non-Redundant Protein Sequences)
**Glycinin G1 (Soy)**		
**FYLAGNQEQEFLK** (qualifier)	✔ ^a^	✔
YQQEQGGHQSQK	✔	✔
NLQGENEGEDK	✔	✔
LNALKPDNR	✔	**☒** ^b^
GQSSRPQDR	✔	**☒**
SQSDNFEYVSFK	✔	✔
**VLIVPQNFVVAAR** (quantifier)	✔	**☒**
**VFDGELQEGR** (qualifier)	✔	✔
FLVPPQESQ	✔	**☒**
LSAEFGSLR	✔	**☒**
**11S Globulin (mustard)**		
ALPLEVITNAYQISLEEAR	**☒**	**☒**
SEAGQVEYWDHNHPQIR	**/**	✔
**VTSVNSYTLPILQYIR** (qualifier)	**☒**	**☒**
GGQQPQLIEEIVEV	**/**	✔
TNANAMISTLAGR	**/**	**☒**
LAQELQNQQDK	**☒**	**☒**
GPFQVVRPPLR	**☒**	**☒**
QAYESEQWR	**/**	**☒**
**FNTLETTLTR** (quantifier)	**☒**	**☒**
THENIDDPAR	**☒**	**☒**
ADIYKPNLGR	**/**	✔
QSLGVPPQVK	✔	✔

^a^ ✔ = specific peptide; ^b^ **☒** = not specific peptide; **/** = no results with 100% of identity.

**Table 4:** Calibration curve parameters, limits of detection (LOD) and quantification (LOQ), expressed as mg_Prot.Tot_/kg_matrix_, of spiked flour, spiked and incurred cookies by considering VLIVPQNFVVAAR as quantifier marker peptide at *m/z* 713.4^2+^ for soy and FNTLETTLTR as quantifier marker peptide at *m/z* 598.3^2+^ for mustard.

Sample	Slope	R^2^	LOD (mg_Prot.Tot_/kg_matrix_)	LOQ (mg_Prot.Tot_/kg_matrix_)
**Glycinin G1 (Soy)**				
Spiked flour	35.3 ± 0.4	0.999	2.8	9.2
Spiked cookies	36.2 ± 0.2	0.999	--	--
Incurred cookies	11.4 ± 0.3	0.997	6.7	22.2
**11S Globulin (mustard)**				
Spiked flour	12.0 ± 0.2	0.999	2.9	9.8
Spiked cookies	11.8 ± 0.7	0.990	--	--
Incurred cookies	9.0 ± 0.2	0.997	4.3	14.2

## Data Availability

The raw data supporting the conclusions of this article will be made available by the authors upon request.

## Supplementary Materials

The following supporting information can be downloaded at: https://www.mdpi.com/article/10.3390/ijms26083891/s1.
