# Supplementary material for: Contamination of Wheat Flour and Processed Foodstuffs with Soybean and Mustard Allergenic Proteins
Source: Int J Mol Sci. 2025 Apr 20;26(8):3891. doi: 10.3390/ijms26083891 (PMC12027982; doi:10.3390/ijms26083891)
Supplement: Supplementary file 1 [file ijms-26-03891-s001.zip › ijms-3580485-supplementary.pdf]

Supplementary material

Contamination of wheat flour and processed foodstuffs by soybean and mustard allergenic proteins

Mariachiara Bianco*[1,2], Domenico De Palma[3], Antonio Pagano[1], Ilario Losito[1,2], Tommaso R.I. Cataldi[1,2], Cosima D. Calvano*[1,2]

[1] Department of Chemistry, [2]Interdepartmental Research Center SMART University of Bari Aldo Moro, via Orabona 4, 70126, Bari, Italy, [3]Food Safety Lab, Via A. Santelia Architetto, 258, 70033 Corato BA, Italia

Number of Figures: 4

Number of Tables: 2

Keywords: allergens, soybean, mustard, mass spectrometry, flour

Authors for correspondence, email: mariachiara.bianco@uniba.it; cosimadamiana.calvano@uniba.it

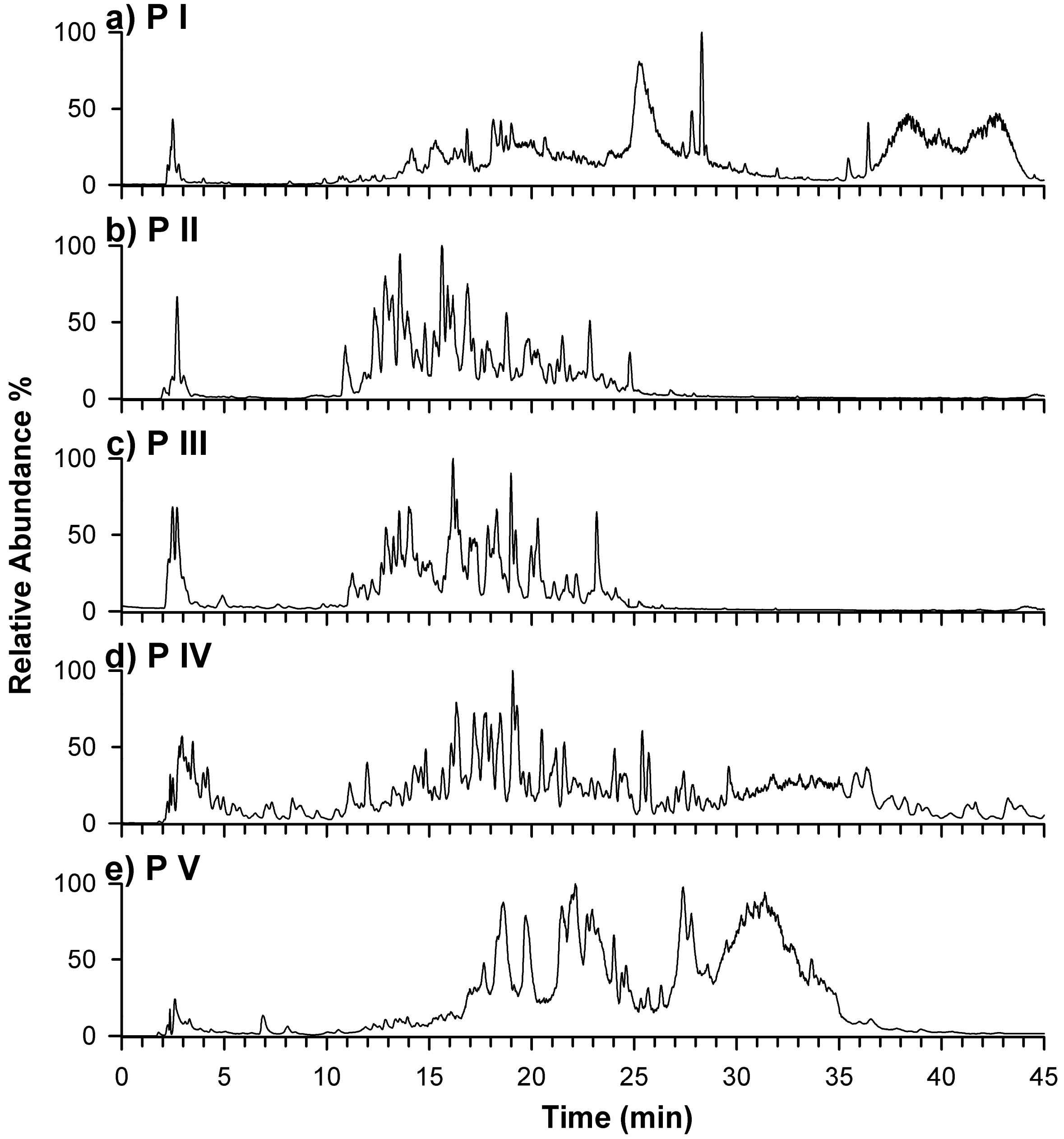

Figure S1. Total ion current chromatograms by RPLC-ESI(+)-FTMS of trypsin digest soybean flour protein using the protocol (**a**) I, (**b**) II, (**c**) III, (**d**) IV, and (**e**) V.

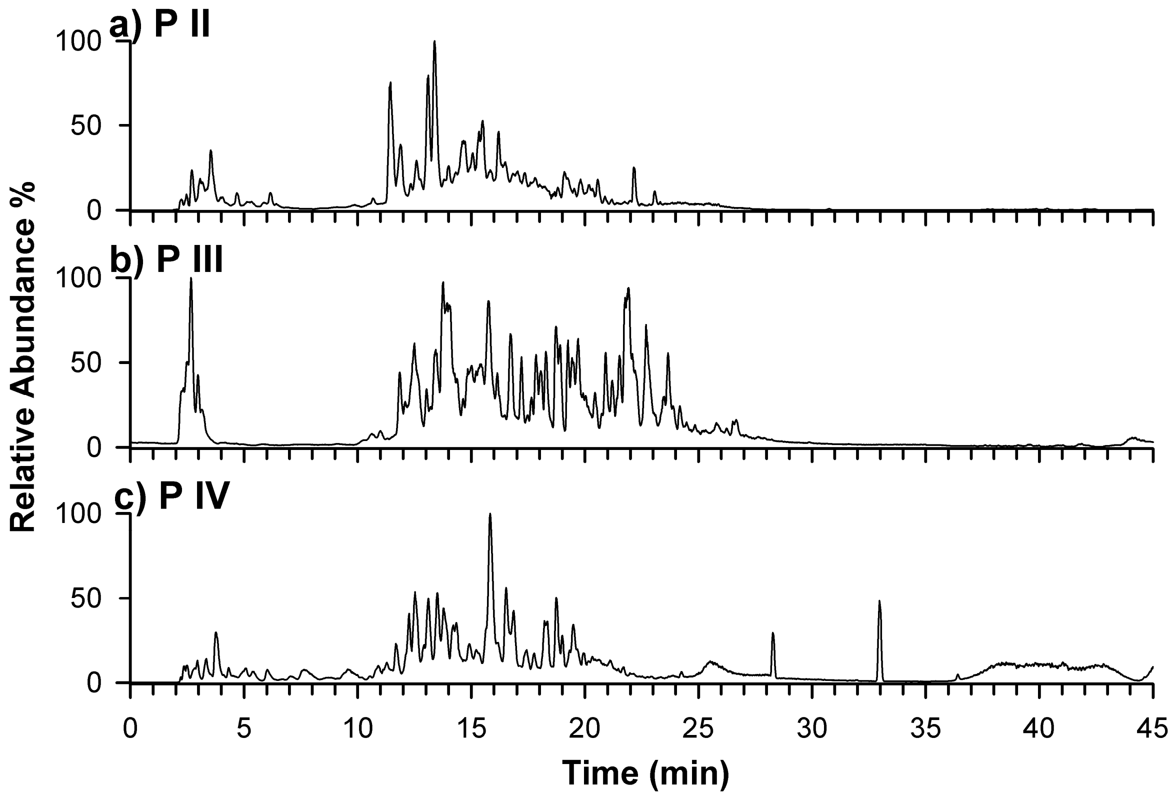

Figure S2. Total ion current chromatograms by RPLC-ESI(+)-FTMS of trypsin digest mustard flour protein using the protocol (**a**) II, (**b**) III, and (**c**) IV.

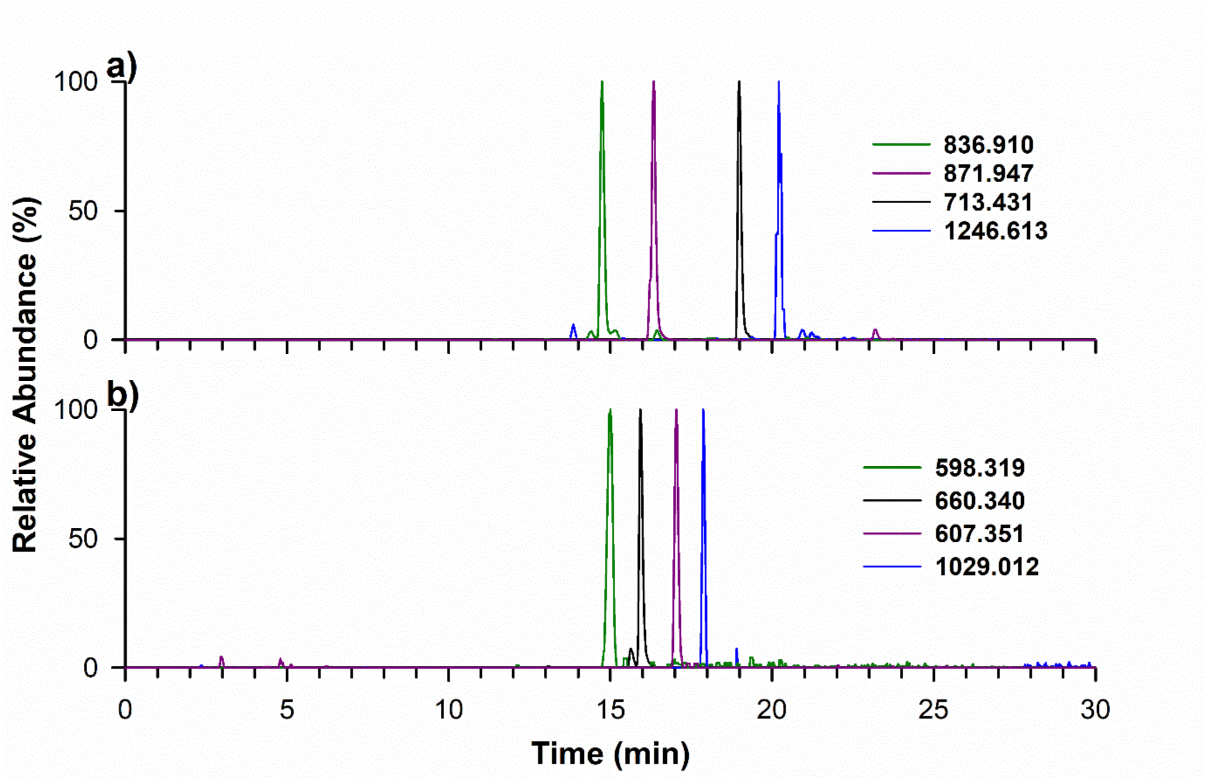

Figure S3. Extracted ion current chromatograms obtained using RPLC-ESI(+)-FTMS for doubly protonated peptides of Glycinin G1 and 11S Globulin. (**a**) Chromatograms for Glycinin G1 peptides at *m/z* 836.910^{2+} (QIAKNLQGENEGEDR), *m/z* 871.947^{2+} (RFYLAGNQEQEFLK), *m/z* 713.431^{2+} (VLIVPQNFVVAAR), and *m/z* 1246.613^{2+} (NAMFVPHYNLNANSIIYALNGR); (**b**) at *m/z* 598.319^{2+} (FNTLETTLTR), *m/z* 660.340^{2+} (TNANAMISTLAGR), *m/z* 607.351^{2+} (GILQGSAMVLPK), and *m/z* 1029.012^{2+} (DACNLDNLDVLQPTEVIK). All peaks were normalized to 100%.

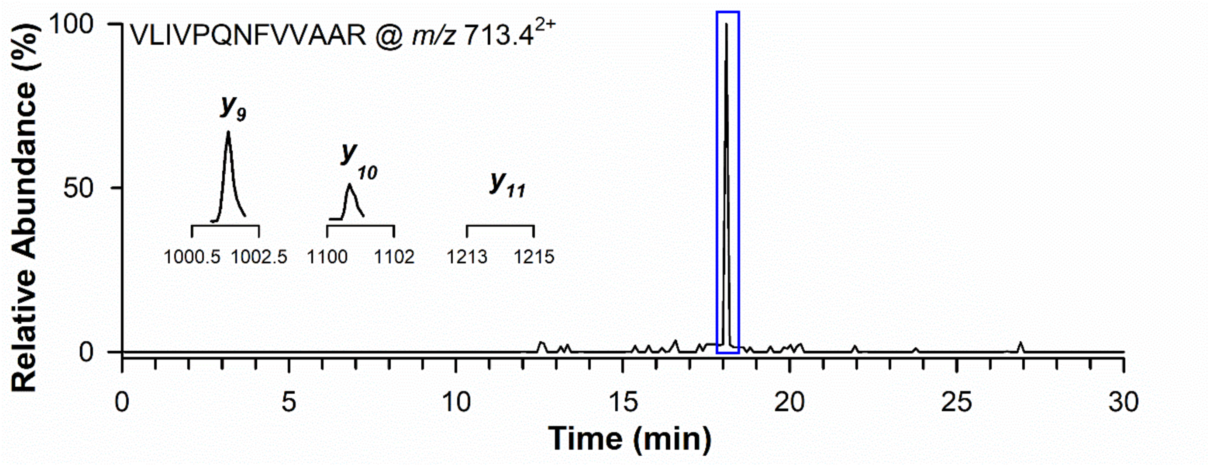

Figure S4. Chromatogram obtained through RPLC-ESI-LIT-MS/MS in the MRM mode illustrating the occurrence of quantifier marker peptide of Glycinin G1 at *m/z* 713.4^{2+}, in cookies made with naturally contaminated wheat flour. In the inserts are the single product ions monitored in the MRM mode.

Table S1. Full list of identified proteins in soy flour sample across all extraction methods.

P I		P II		P III		P IV		P V	
Protein Accession No.	Cov (%)	Protein Accession No.	Cov (%)	Protein Accession No.	Cov (%)	Protein Accession No.	Cov (%)	Protein Accession No.	Cov (%)
I1JLC8	57	I1NGG4	91	Q39805	62	Q9XES8	97	A0A0R0K2T0	52
A0A8H6KX67	53	Q9SEK9	88	P04776	60	I1K7E6	90	I1KZK7	47
A0A0R0HML5	41	P04776	74	P04405	53	Q42447	88	A0A8H6N5P2	46
A0A8H6J0G3	39	I1L849	69	P02858	53	A1KR24	88	I1L3L5	46
A0A8H6N0T9	39	Q9SWB2	69	F8WQS0	49	I1NGG4	88	A0A8H6MNK4	42
Q01527	37	I1LE41	69	F7J077	49	Q9SEK9	87	A0A0R0F2I4	41
A0A0R0G8X6	33	P02858	68	A0A0R0GMV1	45	I2E8L0	80	A0A8H6MQY0	41
Q541U1	31	P04405	67	C6T488	45	A0A0R0FBK4	79	I1JYI8	40
C6SZU1	30	A0A0R0FBK4	67	P11828	43	I1LE41	75	O22378	39
A0A8H6JCW6	30	P11828	64	P01071	41	K7LEQ5	74	A0A1I9LWB7	38
A0A8H6IY75	28	Q7M211	64	A0A8H6KQH9	39	Q9SWB2	69	A0A8H6JY33	37
K7N296	28	A0A0R0GMV1	64	I1NGG4	34	P04776	67	Q9SWB2	37
A0A8H6NC32	27	K7LEQ5	60	P0DO16	33	Q9S7N8	66	A0A8H6JUT2	37
C6SYP2	26	O22121	59	A0A0R0G6E5	32	C6TMA8	66	A0A0R0F963	36
A0A0R0FBK4	26	P0DO16	59	I1L860	30	C6T0L2	62	A0A8H6JN10	33
A0A8H6KIL5	26	A0A0R0L186	58	P19594	30	Q01527	61	A0A8H6NAR6	32
A0A8H6KJA0	25	Q9S7N8	57	P11827	28	K7L505	59	K7MCU2	31
C6TFR3	25	F7J077	56	C6SZ13	28	P04405	59	A0A0R0HZU1	30
C6SZT0	25	Q9XET1	53	C6T0L2	26	I1JLC8	56	A0A8H6J2V5	30
A0A8H6NYT9	24	Q9XET0	53	I1JLC8	25	I1L849	55	A0A8H6U4B9	30
A0A8H6MMC3	24	A1KR24	52	A0A8H6IXN1	24	O23957	54	A0A8H6K413	30
A0A8H6J2V5	24	P11827	52	C6SWW4	24	A0A0R0GMV1	54	A0A0R0I897	29
I1LEL3	23	I1JLC8	50	O22378	22	I1LHR9	51	I1JPZ4	29
A0A8H6ISS6	23	P01071	50	I1N1Y7	21	P0DO16	50	A0A8H6NGX3	29
A0A8H6JUC8	22	O23957	46	C6SX26	21	I1L957	50	C6T1R7	28
A0A8H6KE62	22	Q42447	46	I1N747	19	C6T9C2	50	A0A8H6MW13	28
C6T264	22	C6T488	46	P29531	18	P11828	49	A0A8H6KPW4	28
C6T1Z8	21	I1MQD2	45	Q39858	18	Q7M211	48	C6T340	28
A0A8H6MSA9	21	C6T588	45	P05046	16	A0A8H6KWN2	48	A0A7X4YRA3	28
A0A0R0KKR1	21	C6T0L2	44	I1JF86	16	Q39871	48	K7M5W4	27
A0A8H6NGZ1	21	I1L860	43	C6T1Q7	16	I1MJK6	47	A0A0R0KW87	27
C6SZ52	21	P19594	42	A0A0R4J4U4	15	A0A8H6JS76	47	I1JS10	27
A0A8H6NBV3	20	C6TMA8	41	A0A8H6MIT7	14	Q9XET1	47	A0A8H6NG82	27
A0A8H6IQQ4	20	K7L8B5	39	I1L957	13	C6T488	45	K7LHX2	27
A0A8H6N7M3	20	A0A0R0KVB4	39	I1MQD2	12	Q9XET0	44	A0A8H6NTH0	27
A0A8H6JEB0	20	I1M222	39	B3TDK6	12	P02858	44	A0A8H6NCF0	27
I1MIE5	19	P05046	39	Q01527	11	A0A0R0K7S6	42	K7LVU0	26
A0A8H6NLQ9	19	C6SWW4	38	A0A0R0FBK4	11	Q39801	42	K7M1M8	26

A0A8H6JHI2	19	A0A0R0HE55	38	A0A7X5BVX6	10	I1M222	42	Q01527	26
A0A8H6NDV6	19	I1N1Y7	37			C6T1Q7	41	A0A8H6J833	26
K7LDQ1	19	Q9XES8	35			P19594	41	A0A8H6ITR6	26
K7M7W0	19	A0A8H6IW76	34			F7J077	41	A0A8H6KSQ4	26
A0A8H6NNM3	18	I1JFL5	34			I1JFL5	40	I1L024	26
A0A0R0FE47	18	C6T9C2	34			K7MA69	40	C6SX26	25
A0A7X4YU64	18	I1LVC1	33			Q39873	40	A0A8H6K0Y6	25
C6TJE3	18	I1L957	33			P11827	38	K7LGQ4	25
A0A8H6N4M1	18	I1KYW3	33			F8WQS0	38	I1N980	24
Q9XES8	18	C6T1Q7	33			A0A8H6U4N3	38	A0A8H6NTP5	24
K7L7P7	18	Q9SP11	32			K7MYS4	37	C6TGI0	24
A0A8H6KW35	18	C6TIR2	32			I1N747	34	I1JLC8	24
C3VHQ8	18	P62163	32			I1LVC1	33	A0A8H6J6A7	24
K7LBW6	18	A0A0R0J6X4	31			Q541U1	33	A0A8H6NVF0	23
A0A0R0K7S9	17	Q01527	31			I1M0W1	32	A0A8H6JKA7	23
C6T1Y3	17	B3TDK4	31			A0A8H6NTC2	32	K7N1E3	23
A0A8H6N0V2	17	C6T1V2	29			K7MQN2	32	A0A8H6MTX0	23
A0A8H6MM07	17	O64458	29			C6SWW4	32	A0A8H6KMG6	23
A0A8H6N2N6	17	Q9AVK8	29			K7LJX0	31	A0A3Q7BY30	23
A0A8H6JUA8	17	A0A8H6KKR6	28			K7L2M4	30	I1K7E6	23
O22121	17	A0A8H6JQD4	27			C6T1P5	30	A0A8H6JI37	23
A0A8H6U8I1	17	B3TDK6	25			C3VHQ8	28	A0A8H6KK58	23
A0A8H6MJK5	16	C6SXX2	25			I1KZ37	28	A0A0R0KCY4	22
A0A8H6IN43	16	A0A7X4YW02	25			I1N7C5	28	I1J5H3	22
K7KZE6	16	I1MIA8	25			C6SZ13	28	A0A8H6U4N1	22
A0A8H6KVW2	16	I1JUP4	24			A0A8H6JEG8	28	I1MQD2	22
I1MXZ6	15	C6SVD1	24			C6SZQ6	27	A0A0R0EFK7	22
A0A8H6MJZ7	15	A0A8H6KQU3	24			A0A8H6KKD3	26	A0A8H6K6V8	22
K7MG85	15	B1Q2X4	23			I1N1I4	25	Q541U1	22
A0A8H6KBY4	15	A0A0R0GZI4	23			A0A8H6KS74	25	A0A8H6MZI5	22
K7MW92	15	A0A8H6JAW7	23			K7N2P9	25	A0A8H6KBE3	22
A0A8H6MSJ9	14	O22378	22			A0A8H6MXG1	24	A0A8H6U8Y4	21
A0A8H6KDN6	14	K7L505	22			P29531	24	K7LSZ0	21
A0A0R0JQ91	14	I1JXG9	22			A0A8H6ISS6	23	A0A8H6NF92	21
I1M0F2	14	Q39871	22			C6SZV4	22	C6SVB0	21
A0A8H6KD10	14	A0A8H6J6M6	21			O22378	22	A0A8H6NF00	21
A0A0R0K9Z4	14	C6SX26	21			A0A8H6N8D5	22	A0A8H6JRU2	21
A0A7X4YXL5	14	C6SZ13	21			I1JHY0	22	I1L5F1	20
I1JZS9	14	A0A8H6J7V4	21			A0A8H6MW17	22	A0A8H6JCW6	20
A0A0R0G1C0	14	I1JH03	21			C6TFI3	22	K7MAE3	20
A0A8H6KH29	14	A0A7X4YPM0	21			A0A8H6MWV7	21	A0A0R0EHL2	20
A0A8H6N6U7	14	I1K2F1	21			A0A8H6KR21	21	A0A8H6K4S7	19
A0A8H6IWI0	14	P29531	20			A0A8H6KMT6	21	Q6PV94	19
A0A8H6IUR4	14	I1JZU3	20			I1N1Y7	21	A0A8H6JX08	19
A0A8H6ITY6	14	K7N527	20			A0A8H6J6W4	21	A0A8H6N816	19
A0A0R0L372	14	A0A0R0GJ82	20			A0A8H6K0C7	21	A0A8H6ILR6	19
A0A8H6JG95	14	I1MEE1	20			A0A8H6K0F7	21	A0A8H6N9I7	19

K7L4P0	13	I1K286	20
A0A8H6KCG1	13	A0A8H6NEP5	19
A0A8H6NPN7	13	A0A8H6NBU2	19
C6T588	13	A0A8H6MVT8	19
A0A8H6JFE0	13	Q8RVH5	19
K7LD42	13	K7KTR9	19
A0A8H6JEX0	13	A0A8H6K4R8	19
A0A8H6IX78	13	A0A0R0FW30	19
A0A8H6N5V8	13	C6TBX7	19
K7L3E3	13	A0A8H6ITD6	19
A0A8H6K0V7	13	K7LDQ1	19
A0A8H6IV86	13	A0A8H6KER5	19
I1N747	13	K7LRK8	19
A0A8H6J4P0	13	A0A0R0HYM3	18
A0A0R0EX21	13	I1KZ37	18
C6T457	12	A0A0R0HF66	18
A0A8H6JXB6	12	Q541U1	17
A0A8H6IVN5	12	A0A8H6JWQ5	17
A0A0R0EVK8	12	A0A0R0G014	17
A0A8H6J8Z9	12	A0A0R0FN90	17
A0A8H6JPC4	12	K7MDV2	17
A0A0R0IB46	12	A0A8H6KTA5	17
A0A8H6KUM9	12	I1M3U8	17
C6T283	12	A0A8H6NGC3	17
A0A8H6JQT0	12	A0A8H6JSE8	16
A0A8H6NL98	12	A0A0R0JZP9	16
A0A8H6NVK4	12	A0A0R0F7L8	16
C6SVL2	12	A0A0R0FW25	16
I1JQ07	12	A0A8H6NJ95	16
A0A0R0KY89	11	A0A8H6J6F7	16
C6T1V2	11	Q39873	16
I1JKF4	11	A0A8H6JKZ3	16
C6TK25	11	A0A8H6N7C3	16
Q6IW08	11	A0A0R0LGZ0	16
K7KUW4	11	I1LDH8	16
A0A7X5C182	11	A0A8H6N6E9	16
A0A8H6J9D6	11	Q39801	15
A0A7X4YNQ0	11	A0A0R0HY71	15
K7LY08	11	A0A7X5C096	15
A0A8H6JTZ1	11	A0A8H6NLG7	15
A0A8H6JKS5	11	A0A8H6JKE7	15
I1JMW4	11	K7KPW9	15
A0A8H6JJ22	11	A0A8H6JU83	15
A0A8H6MT51	11	A0A8H6KMC1	15
A0A0R0EDR0	11	A0A8H6NWL3	15
C6TKM2	11	A0A0R0KNB7	15
A0A8H6IQX9	11	O23959	15

A0A8H6JSP9	21	I1N1P6	19
A0A0R0K792	21	A0A0R0GLC2	19
K7KTR9	21	A0A8H6NA90	19
K7LQI7	20	A0A8H6ND80	19
A0A8H6N4M4	20	K7LDQ1	19
P05046	20	K7M1X3	19
A0A8H6J741	20	C6TK63	18
A0A8H6KJN8	20	I1LI08	18
K7MHX3	20	A0A8H6JEE9	18
A0A8H6IVN5	19	A0A8H6KKZ3	18
I1LPN7	19	Q9XES8	18
A0A8H6ILP5	19	A0A8H6NKD8	18
C6TBX7	19	A0A8H6KXB8	18
C6SVT4	19	A0A7X4YV59	18
K7LDQ1	19	A0A8H6MTP2	18
A0A0R0KKP8	19	K7MCQ3	18
A0A8H6KG46	18	C6T0E9	18
A0A0R0L498	18	K7LBW6	18
I1JQD4	18	A0A8H6IRK0	17
A0A8H6IZQ9	18	K7KSE0	17
A0A8H6JEE9	18	C6TF85	17
A0A8H6K0J6	18	A0A8H6K8T0	17
A0A0R0HF66	18	I1LPP2	17
A0A8H6IX78	18	A0A0R0GJK7	17
K7LQA2	18	A0A8H6NF04	17
C6SXY0	18	K7M0E1	16
A0A8H6NWM0	18	A0A0R0JVF5	16
K7MHA6	18	A0A8H6MVI7	16
A0A8H6JZT6	17	C6SZQ6	16
A0A0R0KPA8	17	K7M527	16
I1N0S3	17	I1MN71	16
A0A0R0GJK7	17	A0A8H6J5B3	16
K7K902	17	A0A8H6KA31	16
A0A8H6J4S9	17	C6SYT6	16
A0A0R0GJ82	16	K7L8L5	15
A0A8H6NXB2	16	A0A0R0H3N9	15
A0A0R0FCT0	16	A0A8H6KVG3	15
V9PI24	16	A0A8H6MRA7	15
K7MID0	16	A0A8H6N4Y7	15
A0A8H6IQF6	16	A0A7X5C2C6	15
C6TB17	16	I1LUM8	15
A0A8H6JQE0	16	A0A8H6J6P8	15
C6K8D1	16	A0A8H6NMV3	15
A0A8H6JLP3	15	A0A8H6JGP0	15
C6T0X9	15	I1KEH1	15
I1JMQ3	15	A0A0R0E7R5	15
C6T390	15	A0A8H6ISW5	15

A0A8H6J9L3	11	I1L178	15
A0A0R0LE89	11	A0A8H6MLU4	14
A0A7X4YMV2	11	I1KB09	14
C6SVH0	11	A0A8H6NWY5	14
A0A7X4YQA1	11	K7LHW8	14
A0A0R0KQM5	11	A0A8H6JMY8	14
I1NCE2	11	A0A0R0LIN0	14
A0A8H6JMY3	10	I1KSS4	14
A0A8H6NWE0	10	C6K8D1	14
A0A8H6NSH6	10	A0A8H6JRR8	14
A0A7X4YLN6	10	A0A8H6NSY5	13
C6T4E2	10	M1FIV7	13
A0A8H6MPG4	10	A0A8H6N468	13
I1MGJ5	10	I1MEC8	13
I1M6U2	10	C6TCF1	13
O23959	10	A0A8H6KM74	13
A0A7X5BXV4	10	C6TN45	13
A0A8H6NY60	10	A0A8H6NE26	13
Q9XET0	10	A0A8H6NTL0	13
I1M946	10	A0A8H6KXH9	13
I1L1M1	10	K7N005	13
A0A8H6MRI7	10	A0A8H6MZF7	13
K7LCK5	10	I1MV31	12
I1LGX3	10	A0A8H6KL64	12
I1KL84	10	I1L2K5	12
C6SYH5	10	I1JKT9	12
K7M871	10	K7KC70	12
A0A0R0GRX8	10	A0A0R4J2Q5	12
K7LJ87	10	K7MFC7	12
A0A7X4YRN2	10	I1MCH6	12
A0A8H6KGI5	10	I1N5S0	12
I1J4V3	10	I1N7N9	12
I1L849	10	K7MU72	12
A0A8H6N4F9	10	I1JZ92	12
K7N3M0	10	A0A8H6JSF7	12
		A0A8H6MMK9	12
		A0A8H6NPY5	12
		I1JMQ3	11
		A0A8H6NWW5	11
		A0A0R0GK94	11
		A0A0R0G0Q4	11
		A0A8H6N1P1	11
		I1LHP6	11
		C6THF2	11
		Q9XER5	11
		A0A8H6MJA4	11
		A0A7X5C1K2	11

A0A8H6JTY2	15	A0A0R0KC66	15
I1JD69	15	A0A8H6IXY3	15
A0A0R0F3R8	15	A0A8H6N2X4	15
A0A8H6KUE2	15	A0A8H6IVR6	15
K7LMP8	15	A0A8H6JDX7	15
Q9RHB7	15	A0A0R4J488	15
I1M1J8	15	A0A8H6JHS4	15
A0A8H6IUS5	15	A0A8H6KGC3	15
A0A0R0F8T7	15	P19594	15
A0A8H6MS32	14	K7L2V2	14
A0A8H6J6H0	14	K7MEB4	14
A0A8H6MSJ9	14	B8LFE2	14
A0A8H6JD87	14	C6T4P1	14
A0A0R0HEC8	14	A0A0R0GA88	14
A0A8H6J0K1	14	A0A0R0F8A7	14
K7KWJ4	14	A0A0R0FBK4	14
K7LH04	14	A0A0R0FPK3	14
C6TFZ8	14	I1LAJ0	14
A0A8H6JIG5	14	A0A7X4YPK2	14
A0A8H6NFW2	13	C6T4U7	14
C6T588	13	A0A8H6JG55	14
A0A0R0KCE5	13	A0A0R0J5B2	14
A0A8H6U8Q9	13	A0A7X4YTQ6	14
A0A8H6KNU4	13	A0A8H6IPS3	14
A0A8H6N3M5	13	A0A8H6J1K8	14
A0A8H6JIH4	13	A0A8H6MKC1	14
A0A8H6NB20	13	A0A8H6IPM6	14
C6SWS8	13	A0A8H6K6Y0	14
A0A8H6KCM3	13	A0A0R0GTN8	14
A0A8H6IQY2	12	A0A8H6JCI7	14
A0A8H6NA01	12	A0A8H6JM73	14
A0A8H6KWQ1	12	A0A0R0G3H9	14
K7L6W6	12	I1MYV4	14
A0A8H6KPP7	12	A0A8H6JG95	14
I1MQD2	12	A0A8H6IVN5	14
A0A8H6JS44	12	A0A8H6MXC2	14
Q04672	12	A0A8H6U7U5	14
I1J520	12	I1KDX0	13
C6SX26	12	A0A8H6JSY6	13
C6T283	12	A0A8H6IUP1	13
I1M9R8	12	A0A8H6NZR9	13
K7LBR0	12	A0A8H6KY80	13
A0A8H6U732	12	C6SXM8	13
K7LP51	12	A0A8H6NGX6	13
A0A8H6IY62	12	A0A7X5C3S3	13
A0A368UMV9	11	A0A0R0HLW9	13
A0A8H6N4S9	11	I1LMJ9	13

K7LVF1	11
A0A8H6MZ35	11
I1M1B6	11
A0A8H6J593	11
C6SYC6	11
I1K725	11
A0A8H6J3Q6	11
K7N0X6	11
I1N747	11
A0A0R0JVF5	11
A0A8H6JCW6	11
A0A0R0JV58	11
K7M4I5	11
A0A8H6N0W4	10
I1JSE0	10
P25273	10
A0A8H6IX25	10
I1NAI0	10
K7KLK3	10
A0A0R0FL69	10
I1M3G0	10
A0A8H6NU65	10
I1NHK3	10
A0A8H6JAD5	10
C6T1K1	10
A0A7X4YK60	10
K7LP96	10
A0A0R0KXZ6	10
K7KJK2	10
I1M7J4	10
K7L2S2	10
A0A8H6NPF3	10
K7LGQ5	10
I1JXH6	10
I1L4C8	10
C6TB70	10
K7M2P0	10
A0A8H6KT67	10
A0A8H6JTE3	10

A0A0R0GK94	11	K7L5K3	13
A0A8H6KME0	11	C6T8E5	13
A0A8H6IPH3	11	K7LBM0	13
I1JSQ5	11	I1K4E2	13
K7MQV9	11	A0A0R0GIS8	13
A0A8H6NUG0	11	A0A8H6KPA4	13
A0A8H6JW28	11	A0A8H6IP07	13
I1NCX7	11	A0A8H6NFN7	13
K7LVF1	11	A0A8H6KME2	13
C6TH20	11	A0A0R0FQD3	13
A0A8H6MNW5	11	K7KP96	13
A0A8H6NG11	11	C6SWS8	13
I1JQN4	11	A0A8H6NUA6	13
A0A0R0JX37	11	A0A0R0GE50	13
Q38JD2	11	A0A7X5BX58	13
A0A7X4YJN7	11	C6TK33	13
A0A8H6JD75	11	C6TLG7	13
A0A8H6KW47	11	A0A8H6NN38	12
A0A8H6N6B5	11	A0A8H6J232	12
A0A8H6IYE7	11	C6TA77	12
K7LXM3	11	A0A0R0L867	12
A0A8H6JHA7	10	K7N118	12
A0A8H6JW11	10	A0A8H6N668	12
A0A8H6MNI2	10	A0A8H6J2E5	12
A0A8H6MSA4	10	K7KVY3	12
A0A8H6J658	10	A0A8H6NQH5	12
I1JEB0	10	I1KJR8	12
A0A7X4YPE1	10	A0A8H6NTX9	12
A0A8H6JGK2	10	A0A8H6KUM9	12
C6SVZ0	10	A0A0R0K8P5	12
A0A8H6K9D2	10	A0A8H6J0H2	12
A0A7X5BVQ0	10	I1MV02	12
A0A0R0HBQ4	10	I1MBW2	12
A0A7X4YQH4	10	A0A8H6KWV2	12
A0A8H6JME2	10	A0A7X4YMZ0	12
A0A7X4YSW4	10	A0A8H6K4F5	12
I1KM24	10	A0A7X4YSA1	12
A0A8H6ISG7	10	K7MLG8	12
I1KXT7	10	A0A8H6N4Q9	12
A0A0R0GG92	10	C6T414	12
A0A8H6NFJ7	10	A0A8H6NTS8	12
C6SZ50	10	A0A8H6JCV7	12
A0A7X4YS69	10	A0A8H6MNE4	12
A0A8H6KXT0	10	A0A8H6JXU8	12
I1KJM9	10	A0A0R0JE17	12
C6TGZ4	10	K7LQ43	12
A0A8H6JTU5	10	A0A8H6INU9	11

C6SX87	10	K7LTL8	11
K7KER2	10	A0A8H6N2I4	11
C6TDV6	10	A0A0R0IHU3	11
O23959	10	A0A8H6U942	11
I1J9W0	10	A0A8H6U660	11
A0A8H6IT52	10	C6TCH5	11
A0A0R0HYM3	10	A0A8H6J6D9	11
K7KRM9	10	A0A8H6NFF0	11
K7LA74	10	A0A7X4YU75	11
A0A8H6NED2	10	I1M6P8	11
		A0A8H6N4R4	11
		A0A8H6K2D2	11
		A0A8H6N4Q2	11
		A0A0R0EKE8	11
		A0A8H6NQC2	11
		A0A8H6MUH4	11
		A0A0R0HSU7	11
		A0A8H6JJM4	11
		A0A8H6IZJ4	11
		A0A8H6KTM6	11
		A0A8H6KVI4	11
		I1N978	11
		I1LGX6	11
		A0A8H6J6G0	11
		A0A0R0HT58	11
		C6TNJ6	11
		A0A8H6NN11	11
		I1JTG9	11
		A0A8H6NC06	11
		I1JBA3	11
		A0A7X5BZW0	11
		I1MMM7	11
		C6SVD4	11
		A0A8H6JUT0	11
		A0A8H6MSA2	11
		I1K4G3	11
		A0A8H6NVV5	11
		A0A8H6J5Q3	10
		A0A8H6KUC8	10
		I1NA83	10
		I1KYY3	10
		I1NCR2	10
		A0A0R0EAL4	10
		A0A8H6KDA1	10
		A0A7X4YPG1	10
		A0A8H6KIH2	10
		A0A8H6NLE1	10

A0A8H6N214	10
A0A0R0JZA3	10
A0A0R0FYN5	10
A0A8H6MKN6	10
A0A8H6K8E2	10
I1JHW4	10
A0A8H6MPA9	10
A0A8H6IT27	10
A0A8H6NBI5	10
K7M6T0	10
A0A8H6MJK8	10
A0A8H6NZK6	10
I1M3I7	10
C6SX03	10
A0A8H6MZE0	10
A0A8H6JAT2	10
A0A8H6K501	10
A0A0R0G9K6	10
A0A0R0H4E3	10
A0A8H6JES0	10
I1K6M7	10
A0A8H6K3X0	10
C6T2S1	10
A0A8H6J779	10
C6T0J0	10
C6TJ60	10
A0A8H6MZD6	10
A0A8H6NX19	10
A0A8H6KKC4	10
A0A8H6NXB8	10
K7M080	10
A0A7X4YRM3	10
A0A8H6NFL7	10
C6T2V2	10
A0A8H6J3K1	10
A0A8H6K7U7	10
A0A8H6MPI6	10
K7MF60	10
A0A8H6IMI9	10
A0A8H6J8W6	10
A0A0R0HQ31	10
A0A8H6KPR6	10
I1MQQ0	10
C6T1Q7	10
A0A8H6KYT3	10
A0A7X4YSP9	10
A0A0R0LED1	10

A0A0R0I1S1	10
A0A0R0KXW4	10
A0A0R0KAM7	10
A0A8H6NWU8	10
C6TFH7	10
A0A8H6N4W5	10
A0A8H6KAL6	10
I1L712	10
I1L849	10
I1MUU3	10
A0A8H6MPP0	10
C6TBP5	10

Table S2. Full list of identified proteins in mustard flour sample across all extraction methods.

P II		P III		P IV	
Protein Accession No.	Cov (%)	Protein Accession No.	Cov (%)	Protein Accession No.	Cov (%)
E6Y2L9	89	Q2TLW0	54	A0A6C0M9Q9	98
Q5S6T1	84	Q2TLV9	43	A1YBM2	74
A0A6C0MBG3	60	P83908	39	A0A075C3K7	59
A0A6C0MBF3	59	P15322	37	Q5S6T1	53
Q9XL92	54	E6Y2L9	17	Q9XL92	51
Q2TLW0	50	A0A6C0MC10	12	P10359	43
P13851	47			A0A075C683	42
Q41282	43			A0A075C3K4	41
Q5S6T2	40			U3M9L8	38
Q2TLV9	39			Q41283	37
P15322	39			A0A075C369	37
A0A075C3K2	38			A0A075C3D5	35
A0A075C369	37			Q41282	34
P10359	35			A0A6C0M5Y4	33
U3M9E5	35			Q05G18	31
U3M975	35			A0A075C375	31
A0A075C3K7	34			A0A6C0M900	31
Q05G18	31			A0A6C0MBM1	26
A0A075C358	31			O81345	25
A0A075C372	29			Q41275	25
O81345	29			Q43125	25
A0A6C0M612	29			P04796	25
K9LNN4	29			A0A6C0M6X7	24
A0A075C3D3	28			Q8GZQ1	23
A0A4Y5UKI4	27			A0A075C686	22
Q41274	27			A0A6C0M8Y4	22
A0A6C0M716	26			A0A075C5B7	22
A0A6C0M8Y4	26			A0A075C366	22
A0A075C684	25			P45854	21
A0A6C0M5X5	23			A0A6N0UP52	20
A0A126X3M9	23			A0A126X3M9	19
A0A6C0M6X7	22			Q56NH9	19
A0A075C362	22			A0A076L2C8	19
A0A075C5B7	22			A0A076L1X3	19
U3M945	22			A0A075C3D3	19
A0A6C0M5W1	22			A0A075C3J9	18
A0A075C3E3	22			A0A6C0M5W9	18
A0A6C0M984	22			P09672	18
A0A075C5A6	21			A0A6C0M607	17
A0A6C0MBM1	21			P11594	17
P40115	21			A0A075C3J7	17
A0A0U3AHS1	21			J7HGW1	17
A0A6N0UNB4	20			A0A6C0M612	16

A0A075C582	20
P29738	20
A0A513U434	19
A0A6C0MBZ6	19
A0A481V722	19
A0A6C0M5W8	18
A0A6C0M5V8	18
A0A0U2PLN7	18
A0A8F9SIY0	18
A0A075C3J7	17
A0A219UXT9	17
A0A219UXZ9	17
A0A219UXX8	17
A0A219UXX5	17
W8DLU8	17
A0A075C686	17
A0A481V8J9	17
B9VWI8	16
A0A6C0M607	16
P29737	16
A0A075C3D5	16
U3M8R8	16
A0A6C0MC10	15
H9APB1	15
A0A899IHM3	15
H9ALZ6	15
Q9XQN8	15
A0A6C0M610	15
A0A6G6C402	15
Q9MTH2	15
A0A075C587	15
A0A3G1M798	15
A0A3G1MDI7	15
P46818	15
A0A075C3J4	14
A0A075C3E7	14
A0A075C688	14
A0A8F9WUW1	14
A0A6C0M776	14
Q56NH9	14
A0A076L2C8	14
A0A076L1X3	14
P46819	14
Q43133	14
A0A075C3J9	14
H9AW88	14
U3M9L8	14

P29736	16
P13851	16
A0A075C372	16
A0A6C0M9X0	16
A0A075C690	15
A0A6C0M5W1	15
A0A2P1CZW7	15
P46818	15
A0A6C0M984	15
A0A075C582	14
V9ZBI1	14
P15322	13
A0A075C687	13
U3M945	13
G4V2S8	13
U3M8R8	13
A0A075C3F0	13
A0A0U3AHS1	12
A0A6C0M815	12
A0A076KX75	12
Q19MU9	11
A0A6C0M941	11
Q9THV5	11
I3QFJ5	11
A0A6N0UNB4	11
A0A481V722	11
P50546	11
B9VWI8	11
A0A6C0M716	10
W8DLU8	10
E6Y2M0	10
A0A5E4QGX2	10

P09672	13
A0A6C0M7A6	13
G0X0L3	13
A0A6C0M829	13
H9AUJ0	13
A0A6C0M893	13
A0A075C354	13
A0A6G6C3S5	13
A0A899IH06	12
A0A2P1CZW7	12
Q8VX01	12
A0A6C0M941	12
Q43125	12
A0A6C0M7P3	12
Q19MU9	11
A0A6C0M7K9	11
G4V2M1	11
Q8GZQ1	11
A0A6C0M5W0	11
Q8LP48	11
A0A6C0M7C1	10
E6Y2M0	10
O65887	10
A0A075C3D8	10
A0A076L4M9	10
I3QFJ5	10
A0A6C0M6Z4	10
